# Expression of a Hyperthermophilic Cellobiohydrolase in Transgenic *Nicotiana tabacum* by Protein Storage Vacuole Targeting

**DOI:** 10.3390/plants9121799

**Published:** 2020-12-18

**Authors:** Manuel Benedetti, Valeria Vecchi, Zeno Guardini, Luca Dall’Osto, Roberto Bassi

**Affiliations:** 1 Dipartimento di Medicina Clinica, Sanità Pubblica, Scienze della Vita e dell’Ambiente, Università dell’Aquila, Piazzale Salvatore Tommasi 1, 67100 L’Aquila, Italy; manuel.benedetti@univaq.it; 2 Dipartimento di Biotecnologie, Università di Verona, Strada Le Grazie 15, 37134 Verona, Italy; valeria.vecchi@univr.it (V.V.); zeno.guardini@univr.it (Z.G.); luca.dallosto@univr.it (L.D.)

**Keywords:** hyperthermophilic cellobiohydrolase, plant cell wall, protein storage vacuole, transgenic *Nicotiana tabacum*, plant immunity, saccharification

## Abstract

Plant expression of microbial Cell Wall Degrading Enzymes (CWDEs) is a valuable strategy to produce industrial enzymes at affordable cost. Unfortunately, the constitutive expression of CWDEs may affect plant fitness to variable extents, including developmental alterations, sterility and even lethality. In order to explore novel strategies for expressing CWDEs in crops, the cellobiohydrolase CBM3GH5, from the hyperthermophilic bacterium *Caldicellulosiruptor saccharolyticus*, was constitutively expressed in *N. tabacum* by targeting the enzyme both to the apoplast and to the protein storage vacuole. The apoplast targeting failed to isolate plants expressing the recombinant enzyme despite a large number of transformants being screened. On the opposite side, the targeting of the cellobiohydrolase to the protein storage vacuole led to several transgenic lines expressing CBM3GH5, with an enzyme yield of up to 0.08 mg g DW^−1^ (1.67 Units g DW^−1^) in the mature leaf tissue. The analysis of CBM3GH5 activity revealed that the enzyme accumulated in different plant organs in a developmental-dependent manner, with the highest abundance in mature leaves and roots, followed by seeds, stems and leaf ribs. Notably, both leaves and stems from transgenic plants were characterized by an improved temperature-dependent saccharification profile.

## 1. Introduction

In recent years, the request for sustainable fuels has promoted research toward the use of plant biomass, an abundant source of renewable energy, for production of biofuels. Biological conversion into fermentable sugars requires production of large amounts of microbial Cell Wall Degrading Enzymes (CWDEs), whose expression in planta might provide a low-cost production platform together with a direct contact with their natural substrates—i.e., plant cell wall polysaccharides. The in muro targeting of CWDEs does enhance the hydrolysis of cell wall polysaccharides into fermentable sugars [1] and yet often leads to undesired side effects [2]. Indeed, CWDEs are produced by phytopathogens to dismantle the cell wall integrity, thus supporting the infection process [3] and providing carbon sources for the microbe [4]. Conversely, plants evolved a system of plant pattern-recognition receptors (PPRRs) to promptly perceive CWDEs secreted by pathogens—e.g., by sensing the products of their activity (Damage Associated Molecular Patterns, DAMPs) [5,6,7] or by recognizing specific epitopes in CWDEs [8,9] such as Microbe Associated Molecular Patterns (MAMPs) [10]. For example, a number of endo-xylanases characterized by the glycoside-hydrolase domain GH11 (see http://www.cazy.org/Glycoside-Hydrolases.html for further details on glycoside-hydrolase (GH) families) triggered an activity-independent immune response in plants, through recognition of a conserved 25-residue peptide [11]. Such events mainly occur at the apoplast/outer membrane interface, where PPRRs are located [12]. Even though the defence responses protect plants against microbial infections, a hyperactivation of immunity response negatively impacts the plant development, likely due to the growth–defence trade-off [6,13]. Therefore, the uncontrolled in planta expression of CWDEs may result into developmental alterations and reduced productivity [14]. The constitutive expression of cellulases in *N. tabacum* resulted in a wide range of enzyme yields and plant phenotypes, depending on (i) the expression strategy adopted, (ii) the substrate specificity and (iii) the catalytic domain of the expressed enzyme [15,16,17,18,19,20,21].

In this work, we explored the possibility of expressing CWDEs in crops by a novel expression strategy. To this aim, the cellobiohydrolase (CBH) portion of cellulosome CelB from the hyperthermophilic bacterium *Caldicellulosiruptor saccharolyticus*, formerly known as CBM3GH5 [22], was constitutively expressed in *N. tabacum* by targeting the enzyme to the protein storage vacuole (PSV) which is an organelle developed ad hoc for protein storage [23]. In plant cells, PSVs and lytic vacuoles (LVs) are distinct organelles which are served by distinct transport vesicles. Accordingly, their membranes are marked by the presence of distinct tonoplast intrinsic proteins [24] with both storage and defence proteins stably accumulating in PSVs at high levels. Interestingly, the C-terminal Pro-Peptide (CTPP) of Chitinase 1 from *N. tabacum* (NtChitinase1), a protein involved in plant defence against fungi [25], was shown to be sufficient for efficient redirecting an apoplast-targeted protein to the PSV [26,27]. Therefore, the CTTP sorting signal from NtChitinase1 was fused to the sequence encoding the highly thermostable CBM3GH5 and transformed into tobacco in order to generate a transgenic plant in which the transgenic enzyme activity was controlled by both the cell compartmentalization and the temperature. The effect of this transgenesis strategy was compared to that of expressing CBM3GH5 with a target signal to the apoplast as for enzyme yield and the impact on the plant fitness.

## 2. Results

### 2.1. Design and Transient Expression of CBM3GH5-HA and CBM3GH5-HA-VAC in N. tabacum

**Figure 1:**
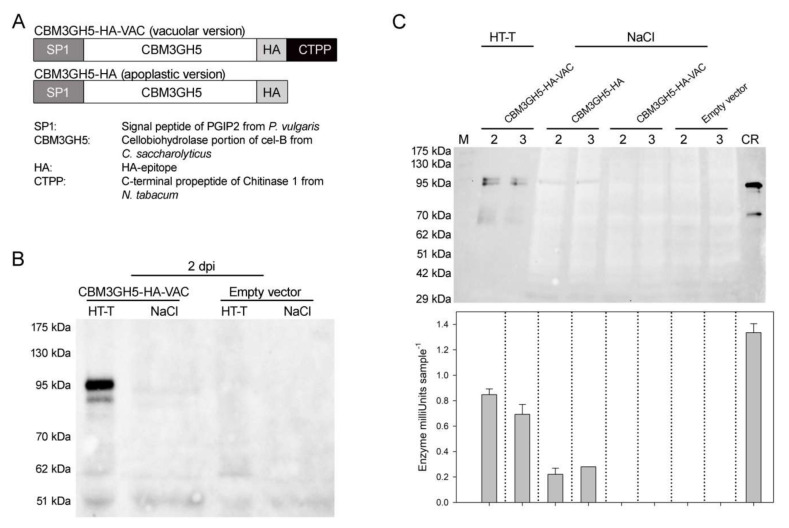
Transient expression of CBM3GH5 in *Nicotiana tabacum*. (**A**) Schematic representation of the vacuolar and apoplast version of CBM3GH5, referred to as CBM3GH5-HA-VAC and CBM3GH5-HA, respectively. Expected molecular weights: 80 kDa. (**B**) Immuno-decoration analysis of leaf extracts from CBM3GH5-HA-VAC-agroinfiltrated plants, using α-HA as primary antibody. Extraction was performed 2 days post-agro-infiltration (dpi) using a Tween 20-supplemented buffer plus heat (HT-T) or a NaCl-supplemented buffer (NaCl). Agroinfiltration with the empty vector was used as negative control. (**C**) (Upper panel) α-HA immuno-decoration analysis of leaf extracts from agroinfiltrated leaves. Extraction was performed two and three days post-agro-infiltration (2, 3) using the same buffers described in (**B**). In total, 70 ng of recombinant CBM3GH5-HA from *C. reinhardtii* (CR) was used as reference. (Lower panel) Activity of CBM3GH5 in the same extracts, determined by activity assay and expressed as Enzyme milliUnits (nmol reducing ends released from carboxy-methylcellulose (CMC) min^−1^) per sample.

In order to target the recombinant protein to the PSV, the CBH portion of cellulosome CelB from *Caldicellulosiruptor saccharolyticus*, previously known as CBM3GH5 [22], was codon-optimized for the nuclear expression in *N. tabacum* and fused at the C-terminus to the sequence encoding the HA-epitope (-YPYDVPDYA--) and the C-terminal Pro-Peptide of Chitinase 1 from *N. tabacum* (--GNGLLVDTM) [26,27,28]. Additionally, the protein CBM3GH5 was capped with the sequence encoding the signal peptide of PGIP2 from *Phaseolus vulgaris* (MTQFNIPVTMSSSLSIILVILVSLRTALSE-) for targeting the enzyme to the apoplast [6,14,29] since the entry in the secretory pathway is required for an efficient vacuole sorting [26,30]; the CBM3GH5-encoding sequence devoid of the C-terminal Pro-Peptide was used to redirect the enzyme to the apoplast (Figure 1A). Vacuolar and apoplastic versions of CBM3GH5 were named as CBM3GH5-HA-VAC and CBM3GH5-HA, respectively. The entire gene sequences used to generate the fusion proteins CBM3GH5-HA-VAC and CBM3GH5-HA are reported in Data S1. Expression of CBM3GH5 was performed in *N. tabacum* as an example of crop plant whose scraps can be used in the bioethanol industry. At the beginning, transient expression in *N. tabacum* was employed to validate the functioning of both the constructs. At 2 days post-infiltration (2 dpi), CMB3GH5-HA-VAC was detected in leaves by immuno-decoration analysis. CBM3GH5-HA-VAC was efficiently extracted from leaf tissue by using heat (70 °C) and a Tween 20-supplemented buffer (0.4% *v/v*), while NaCl-supplemented buffer (0.8 M) failed at extracting CBM3GH5-HA-VAC (Figure 1B). CBM3GH5-HA-VAC was detected as a doublet at molecular weight around 90 kDa in SDS-PAGE gels. Agroinfiltration with the empty vector was also performed as a control. Unlike CBM3GH5-HA-VAC, the apoplastic version of CBM3GH5 was extracted as a single 90 kDa band by NaCl-supplemented buffer (Figure 1C). In these experiments, the chloroplast-expressed CBM3GH5-HA from *C. reinhardtii* was used as reference (Figure 1C) [31]. Both the CBM3GH5 isoforms expressed in tobacco showed a slightly higher molecular weight than the recombinant enzyme from *C. reinhardtii*, suggesting either an event of glycosylation or a partially processed protein. Since the chloroplast translational machinery cannot introduce glycosylation, we expect the algal-expressed enzyme is not glycosylated. All these results suggest that CBM3GH5-HA was expressed as secreted protein, while CBM3GH5-HA-VAC was retained in the cell since the combined use of nondenaturing detergent and heat was required for efficient extraction. Activity assays carried out on the leaf extracts confirmed the presence of the enzyme in the same samples which were shown to contain cross-reactivity towards the α-HA probe: the enzymatic activity was proportional to the relative abundance of the immunotitration signals (Figure 1C).

### 2.2. Stable Expression of CBM3GH5-HA and CBM3GH5-HA-VAC in N. tabacum

**Figure 2:**
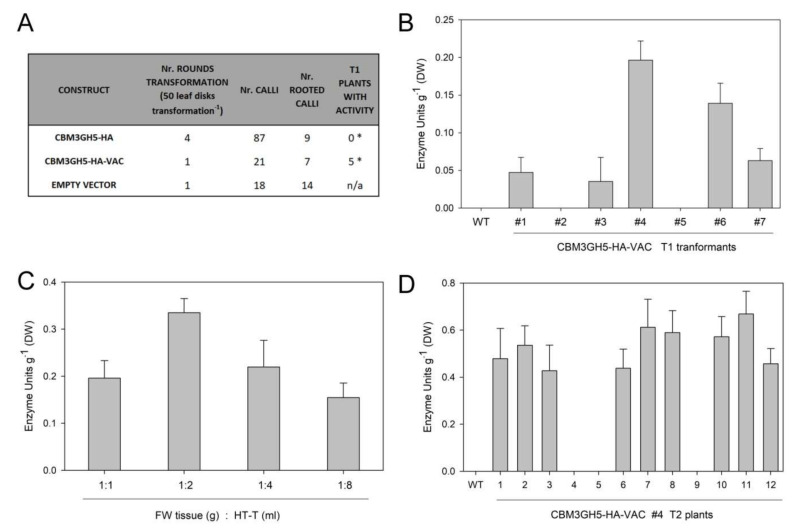
Stable transformation of CBM3GH5 in *Nicotiana tabacum*. (**A**) Comparison of the transformation efficiency of different constructs on *Agrobacterium*-mediated *N. tabacum* transformation. Numbers of regenerated plants and activity in T1 transformants are reported. The association between groups (N rooted calli and T1 plants with activity) and the CBM3GH5 version is statistically significant according to Fischer’s exact test (* *p* value < 0.05). n/a, not available. (**B**) Activity of CBM3GH5 in leaf extract from 30-day-old T1 CBM3GH5-HA-VAC plants, as determined by activity assay. (**C**) Activity of CBM3GH5 in leaf extracts from 30-day-old T1 CBM3GH5-HA-VAC#4 using different ratios of Tween 20-supplemented buffer per gram of tissue. (**D**) Activity of CBM3GH5 in leaf extract from 30-day-old T2 CBM3GH5-HA-VAC#4 plants as determined by activity assay. Enzyme activity is expressed as Enzyme Units (µmol reducing ends released from CMC min^−1^) per gram dry weight (DW) leaf.

Agrobacterium-mediated transformation of leaf tissue was used to generate stable tobacco plants constitutively expressing CBM3GH5-HA and CBM3GH5-HA-VAC. As revealed by transformation efficiency analysis, the isolation of transgenic lines constitutively expressing CBM3GH5-HA was not possible (Figure 2A). Although the presence of the transgene in the genome was confirmed by PCR in the regenerated plants (Figure S1), both gene expression and enzyme activity were not detected in such lines (data not shown), thus suggesting gene-silencing events in the antibiotic-resistant plants [32]. This result is consistent with the evidence [6,14] that apoplastic accumulation of CWDEs may compromise plant development, since the cell wall fragments derived from the residual hydrolytic activity [7,33,34] as well as CWDEs themselves behave as powerful elicitors of plant defence [8,35,36]. It is worth noting that such recognition events occur in the apoplast—namely, the compartment to which CBM3GH5-HA was targeted [10]. Instead, five independent T1 CBM3GH5-HA-VAC-expressing plants were successfully identified by the activity assay performed on 1-month-old transgenic plants (Figure 2B). This result is consistent with CBM3GH5-HA-VAC being retained in the symplast, thus avoiding the detrimental effect that CBM3GH5 exerted in the apoplast. The ratio of nondenaturing extraction buffer per gram of fresh weight (FW) leaf tissue was optimized, and (2 mL extraction buffer: 1 g FW leaf) was selected as the ratio which allows the highest CBM3GH5-HA-VAC recovery yield (Figure 2C). The five independent expressing transformants were grown for seed production and stable plant selection. After segregation analysis, CBM3GH5-HA-VAC#4 and #6 were identified as 3:1 segregating lines. Activity assay performed on 1-month-old T2 CBM3GH5-HA-VAC#4 plants revealed that the enzyme content ranged from 0.4 to 0.7 U g DW^−1^, thus suggesting the homozygous condition increased the CBH content (Figure 2D). CBM3GH5-HA-VAC#4-11 and #6-7 were selected as two independent single-insertion homozygous lines and used for subsequent characterization.

### 2.3. Spatial and Temporal Accumulation Pattern of CBM3GH5-HA-VAC in Transgenic Plants

**Figure 3:**
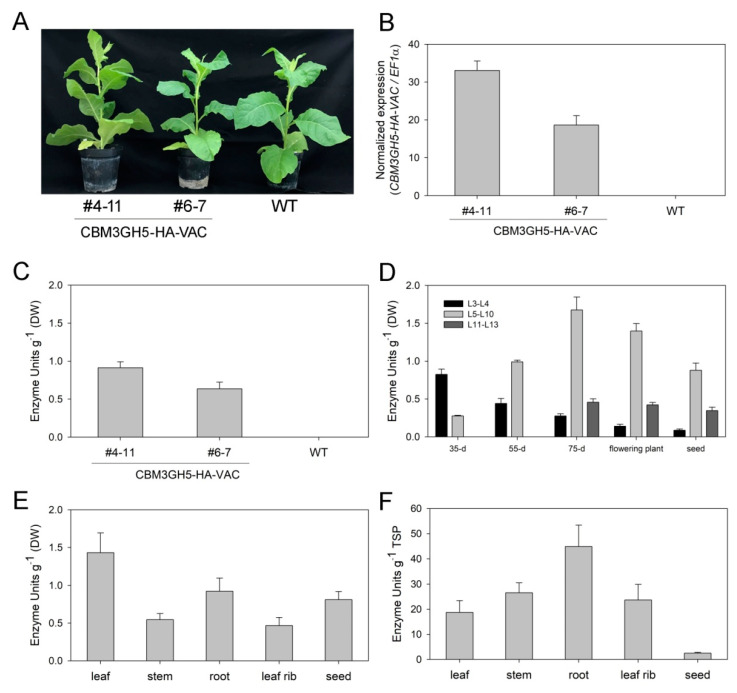
Spatial and temporal accumulation of CBM3GH5-HA-VAC in T3 transgenic plants. (**A**) Representative picture of 55-day-old CBM3GH5-HA-VAC#4-11, CBM3GH5-HA-VAC#6-7 and wild-type (WT) plants. (**B**,**C**) Relative expression (**B**) and activity (**C**) of CBM3GH5 in leaves (L5–L10) from 55-day-old CBM3GH5-HA-VAC#4-11 and #6-7 plants. (**D**) Activity of CBM3GH5 in leaf extracts from CBM3GH5-HA-VAC#4-11 at five different developmental stages (35-d: 35-day-old plant, 55-d: 55-day-old plant, 75-d: 75-day-old plant, flowering plant and seed—i.e., plant upon seed maturation). (**E**,**F**) Activity of CBM3GH5 in different tissue extracts from flowering CBM3GH5-HA-VAC#4-11 plants (i.e., L5–L10 leaf, stem, root and leaf rib) plus seeds per gram DW (**E**) and per gram of total soluble proteins (TSP) (**F**); data are from two independent experiments with consistent results.

CBM3GH5-HA-VAC-expressing plants grew without morphological defects or significant loss in biomass with respect to the control genotype, thus implying that PSV expression of CBM3GH5 did not affect plant development (Figure 3A). Gene expression and enzyme activity analyses were performed on leaves L5–L10 from 55-day-old CBM3GH5-HA-VAC #4-11 and #6-7 transgenic plants (Figure 3A). Both gene expression (Figure 3B) and enzymatic activity (Figure 3C) were higher in the #4-11 than in #6-7 transgenic line, in accordance with the previous result (Figure 2B) with line #4-11, showing 45% higher activity with respect to #6-7 plant (Figure 3C). Therefore, CBM3GH5-HA-VAC #4-11 plant was used to investigate the spatial and temporal distribution of CBM3GH5-HA-VAC during plant development. The expression of a transgene under control of the constitutive 35S promoter is expected to be homogeneous through the different plant tissues. Indeed, tracking the constitutive expression of the Green Fluorescent Protein (GFP) in transgenic tobacco revealed that GFP was uniformly distributed in all the organs, with slightly higher abundance in vascular tissue, whereas it was absent in seeds [37]. However, protein targeting to PSV may alter the expected spatial and temporal distribution of a constitutively expressed protein. In order to investigate this point, the activity of CBM3GH5 was measured during plant development. CBM3GH5 activity in leaves reached a maximum level at the preflowering and flowering stages (Figure 3D). The highest activity was observed in the most expanded leaves of each developmental stage, while the activity was lower in young leaves and gradually decreased proceeding from mature to senescent leaf (Figure 3D). Thus, the expression of CBM3GH5-HA-VAC was dependent on a developmental regulation consistent with a previous report for a recombinant protein targeted to the LVs [38]. As additional information, the activity of CBM3GH5 was evaluated in different organs of the flowering plant: the higher level of activity was detected in leaves, roots and seeds, while activity was significantly lower in stem and leaf ribs (−60% than leaves, Figure 3E). Therefore, the spatial analysis of cellulolytic activity supported the vacuole sorting of CBM3GH5-HA-VAC, since PSVs have been reported to accumulate in seeds and storage organs such as roots [39]; it is noteworthy that the evaluation of enzymatic activity in the different plant organs suggested a mapping of PSV distribution, pointing to the presence of PSVs in the root apparatus of *N. tabacum*. Notably, the highest abundance of CBM3GH5 activity per gram of total soluble proteins (TSP) was detected in roots in accordance with the TSP content of seed (200 mg TSP g DW^−1^), leaf (70 mg TSP g DW^−1^), stem and root extracts (18 mg TSP g DW^−1^) (Figure 3F).

### 2.4. Biochemical Characterization of CBM3GH5-HA Purified from the Transgenic Plants

**Figure 4:**
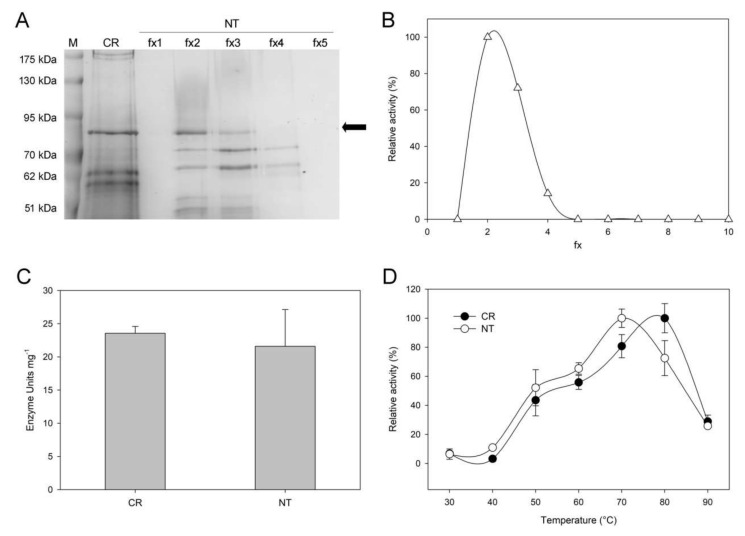
Biochemical characterization of CBM3GH5-HA-VAC purified from mature leaves of CBM3GH5-HA-VAC#4-11 plants. (**A**) SDS-PAGE analysis of fractions (fx) eluted from anionic exchange chromatography (AEC). Black arrow points to CBM3GH5-HA. Recombinant CBM3GH5-HA from *C. reinhardtii* (CR) was used as reference. (**B**) Activity of CBM3GH5-HA-VAC in the same fractions (fx) shown in (**A**), expressed as relative activity (%). (**C**) Specific activity of CBM3GH5 from *C. reinhardtii* (CR) and *N. tabacum* (NT) towards 1% CMC; specific activity was expressed as Enzyme Units per mg of enzyme (pH 5.5, 75 °C). (**D**) Relative activity (%) of CBM3GH5 from *N. tabacum* protein storage vacuole (NT) and *C. reinhardtii* chloroplast (CR) towards 1% CMC, after 7 h incubation at different temperatures.

Different from evidence of transient expression (Figure 1), CBM3GH5-HA-VAC could not be detected by immuno-decoration analysis in stable transgenic plants (data not shown) despite the enzymatic activity being clearly detectable (Figure 2 and Figure 3). This evidence could be ascribed to the maturation process of CBM3GH5-HA-VAC upon PSV sorting. We hypothesize an unpaired cleavage of the C-terminal Pro-Peptide, affecting the integrity of HA-epitope, thus compromising detection by α-HA. In order to further characterize the expression of the recombinant enzyme, we purified CBM3GH5-HA-VAC from mature leaves of transgenic *N. tabacum* and performed a biochemical characterization. Purification was performed by a two-step procedure consisting of a heat-mediated enrichment of the total protein extract [40] followed by an anionic exchange chromatography (AEC) [22,31]. Extraction was carried out by using both the Tween 20-supplemented buffer and heat, following the optimized conditions of Figure 2C. The elution of CBM3GH5-HA-VAC from AEC was performed by a stepwise NaCl gradient. The activity co-eluted with a single 80 kDa band as revealed by enzymatic assay and SDS-PAGE analysis (Figure 4A,B), thus confirming that CBM3GH5-HA-VAC was expressed in mature tobacco leaves, displaying the same molecular weight of the recombinant CBM3GH5-HA from *C. reinhardtii* [31] (Figure 4A). The evaluation of specific activity towards 1% carboxy-methylcellulose (CMC) revealed that both plant and microalgal versions of the enzyme showed similar specific activities (Figure 4B); the activities at different temperatures upon prolonged reaction time also revealed similar enzymatic properties of the two enzymes (Figure 4C). Despite both preparations being hyperthermophilic, the enzyme produced in microalgae showed a temperature-dependent peak shifted towards higher temperature by 10 °C. Treatment of the eluted protein from AEC with PNGase A did not alter the electrophoretic mobility, which was the same in the isoforms expressed in plant and in alga (Figure S2), thus suggesting the recombinant protein was not modified by *N*-glycosylation. Together, these results suggest that the fusion of SP1 and CTPP at the N- and C-termini of CBM3GH5, respectively, allowed the vacuole compartmentalization of CBH in a non-*N*-glycosylated form.

### 2.5. Leaves and Stems from CBM3GH5-HA-VAC-Expressing Plant Showed an Increased Temperature-Dependent Saccharification Yield

**Figure 5:**
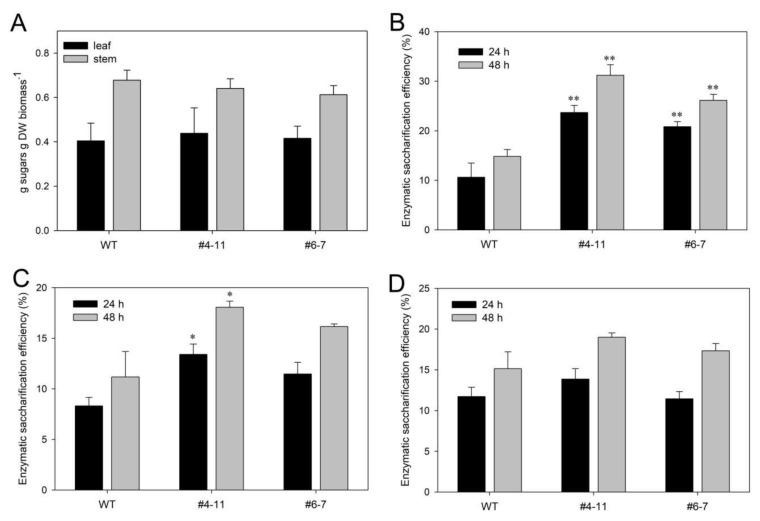
CBM3GH5-HA-VAC plants showed an increased temperature-dependent saccharification efficiency. (**A**) Sugars released upon acid-hydrolysis of leaf (black bar) and stem (grey bar) material from wild-type and transgenic CBM3GH5-HA-VAC plants, as determined by phenol–sulfuric acid assay. (**B**,**C**) Saccharification efficiency of leaf (**B**) and stem (**C**) material from CBM3GH5-HA-VAC#4-11, CBM3GH5-HA-VAC#6-7 and WT plants after 24 (black bar) and 48 (grey bar) hours of incubation with 1% Celluclast at 55 °C. (**D**) Saccharification efficiency of leaf material from CBM3GH5HAVAC#4-11, CBM3GH5HAVAC#6-7 and WT plants after 24 (black bar) and 48 (grey bar) hours of incubation with 1% Celluclast at 25 °C. Data are expressed as mean ± SD (*N* ≥ 3). Asterisks indicate statistically significant difference against control (WT) according to Student’s t test (* *p* < 0.05; ** *p* < 0.01).

Plant expression of CWDEs can be used for generating plants with enhanced saccharification efficiencies. To evaluate if the vacuole accumulation of CBM3GH5 affected the sugar release from *N. tabacum* cell walls, saccharification efficiency was assessed on stems and leaves with the highest enzyme contents (i.e., L5–L10), and expressed as the ratio (%) between solubilized reducing sugars and total sugars in the starting tissue [41]. Determination of total carbohydrates of tobacco leaves and stems confirmed that both wild-type and transgenic plants had the same sugar content (Figure 5A).

In the saccharification experiment, the commercial cellulolytic blend Celluclast was supplemented in the incubation medium at mid-high temperature (55 °C), since Celluclast retains ~95% of starting activity after a 1-day incubation at 50 °C [42]; thus, treatments at 55 °C are a good compromise between the activity of the commercial cellulolytic blend and CBM3GH5 specific activity vs. temperature [22]. The saccharification efficiency of leaf and stem biomass from transgenic lines #4-11 and #6-7 was 13% to 16% (as absolute values) higher than wild-type samples (Figure 5B,C), thus indicating that CBM3GH5 synergistically acted in supporting the cellulolytic activity of the commercial blend. The release of reducing sugars was proportional to the activity of CBM3GH5-HA-VAC as indicated by the increased release in the higher-expressing CBM3GH5-HA-VAC plant—i.e., #4-11; interestingly, the increased enzymatic saccharification of CBM3GH5-HA-VAC line #4-11, with respect to line #6-7, was statistically significant towards stems—i.e., a highly hydrolysis-recalcitrant material (Figure 5C). On the opposite side, at the lower temperature (25 °C), saccharification efficiency of transgenic plants decreased at the same level as wild-type plant (Figure 5D), thus indicating that the release of sugars was temperature-dependent, and suggesting that no major changes in the cell wall architecture occurred in the transgenic lines. As expected from the synergistic action between Celluclast and CBM3GH5, the reducing sugars released from leaf material upon 48 h of incubation were mainly constituted by glucose (i.e., 65–70%)—i.e., the monosaccharide constituting cellulose (Figure S3).

## 3. Discussion

Plant cell wall structures can be successfully altered by using focused transgenic-based strategies for increasing the saccharification efficiency of plant biomass: alterations in cell wall structures may be achieved by either modifying the level of endogenous enzymes involved in the metabolism of plant cell wall polysaccharides or accumulating recombinant CWDEs into the apoplast. The outcome of such strategies is not easily predictable, resulting in a wide range of different plant phenotypes. For example, transgenic plants with altered pectin structures or with altered hemicellulose and lignin contents [43,44] resulted in higher enzymatic saccharification, as expected by the more relaxed cell wall matrix which favored the accessibility of cellulolytic enzymes to cellulose fibers [41]; however, the alteration of cell wall structures resulted in altered phenotypes and morphological defects [14]. In other cases, the altered expression of endogenous enzymes of cell wall synthesis resulted in both higher saccharification and biomass productivity, as observed by downregulating the expression of the galacturonosyl-transferase 4 (*GAUT4*)-encoding gene in switchgrass and poplar [45]. Biological conversion by CWDEs is limited by high costs and low efficiency, and should be compared to chemical methods which are harmful for the environment. The cost-effectiveness of microbial CWDEs and Cell Wall Modifying Enzymes (CWMEs) produced in transgenic plants depends on enzymatic activity and level of accumulation in the plant tissue; however, avoiding growth defects related to the CWDE carbohydrate-degrading activity remains a major challenge. In order to circumvent such undesired phenotypes, plant expression of CWDEs can be controlled by ad hoc expression strategies [2,15]. In this regard, compartmentalized expression was attempted to selectively accumulate CWDEs in organelles such as chloroplasts [16,17], LVs [18] or in the cytoplasm [19,20], thus avoiding the interaction with the cell wall polysaccharides and PPRRs. It is worth noting that N- and O-linked glycosylation can be critical for stability of certain fungal cellulases [46], and since these post-translation modifications mainly occur in endoplasmic reticulum (ER) and Golgi apparatus, they cannot be operated on plastid-expressed enzymes. Although delivery of recombinant proteins to LVs was successfully achieved [47], vacuole sorting of CWDEs was dependent on the development of LVs in each plant tissue [38]. Control of CWDE-encoding gene expression was attempted by using synthetic promoters induced by ethanol [48] and β-estradiol [49] or by endogenous pathogen-induced (e.g., *PR-1* (At2g14610), *RetOx* (AT1G26380)) [6] and senescence-induced promoters (e.g., *SAG12* (AT5G45890)) [50]. However, endogenous promoters are often affected by transcriptional leakiness [6] or are not ideal for supporting a robust transgene expression [50]. Last but not least, by expressing enzyme isoforms with high (>70 °C) temperature-dependent activities, such as those from hyperthermophiles, the activity of CWDEs can be quenched during plant development [51,52].

In our study, the thermostable cellobiohydrolase CBM3GH5 was expressed in transgenic *N. tabacum* plants by targeting the enzyme to the PSV. When developing a technology for engineering plants producing CWDEs, the choice of a highly thermostable CBH has several advantages: (i) the low activity of CBM3GH5 at the growth-temperature of *N. tabacum* should prevent side-effects on plant health, in the event PSV sorting may suffer for leakiness; (ii) the heat treatment can promote both the release of the enzyme from PSV as well as the relaxation of the polysaccharide matrix, further increasing hydrolyzation efficiencies [53]; (iii) the temperature-dependent activity of CBH can be used to pinpoint the enzyme in the different organs during plant development. The latter point is crucial having been the first attempt, to our knowledge, of targeting a recombinant protein to PSV in a crop species; (iv) CBHs, together with β-glucosidases, are key-enzymes for the degradation of cellulose [2,15]. In this regard, the presence of carbohydrate binding module (CBM) in CBM3GH5 was expected to enhance the hydrolysis of crystalline cellulose [54,55].

In order to pursue this issue, CBM3GH5 was overexpressed by either apoplast or PSV targeting, and the effects of the two different strategies were investigated. Surprisingly, we failed to isolate stable transgenic lines expressing the apoplastic version of CBM3GH5, although the transient expression analysis confirmed the apoplast-targeted enzyme was efficiently translated (Figure 1C). The lack of stable CBM3GH5-expressing transformants was statistically significant, pointing to the apoplastic localization of the hydrolase as a negative selection trait in tobacco plants (Figure 2A). This result was unexpected since the hyperthermophilic nature of CBM3GH5 should, in principle, prevent any cellulolytic activity at the growth temperature of tobacco, thus preserving plant fitness. To explain the potential “lethal” phenotype, it seems conceivable that in muro CBM3GH5 expression exerts its deleterious effects by a residual hydrolyzing activity at greenhouse temperature (Figure 4D). Alternatively, the recombinant enzyme could be recognized as a MAMP by tobacco PPRR, thus affecting plant development. According to this hypothesis, expression of GH5 cellulases resulted in plants with morphological defects or with low cellulolytic activities, despite the enzymes being compartmentalized or endowed with a temperature-inducible activity [17,31,56]. Alternatively, the lack of stable transformants accumulating CBM3GH5 in the apoplast could be a consequence of a reduced stability of CBM3GH5-HA with respect to CBM3GH5-HA-VAC. Such reduced stability may be ascribed to endogenous proteolysis, which is expected to lack in ad hoc compartment evolved to store proteins—i.e., the PSV. Additionally, unwanted glycosylation may affect enzyme stability; indeed, opposite to that expected for the apoplastic isoform, CBM3GH5-HA-VAC was expressed in a nonglycosylated form, which appears favorable since unnecessary glycosylation could impair the stability of bacterial enzymes (Figure 4A and Figure S2). Based on the specific activity of tobacco-expressed CBM3GH5-HA-VAC, the yield of the enzyme in PSV was about 0.08 mg g^−1^ DW in the mature leaves of *N. tabacum* (see Figure 3D and Figure 4B: yield calculated as (1.67 U g DW^−1^)/(21.59 U mg^−1^)) whereas the percentage abundance ranged from 0.09% to 0.2% of TSP in leaf and root tissues, respectively (see Figure 3F—i.e., percentage abundance in leaf extract calculated as (18.7 U g TSP^−1^)/(21,590 U g^−1^) × 100%; percentage abundance in root extract calculated as (44.8 U g TSP^−1^)/(21,590 U g^−1^) × 100%). As observed for expression in LVs, the plant developmental stage is crucial for accumulation of CBM3GH5-HA-VAC [38]: the highest level was detected in the most expanded leaves of the preflowering and flowering stages while a 50–80% reduction was measured in senescent leaves (Figure 3D). Interestingly, a relevant activity of CBM3GH5 was also detected in roots and seeds, indicating PSV sorting as an additional tool for accumulating recombinant proteins in bulb-plants and seeds (Figure 3E,F).

Notably, PSV sorting of CBM3GH5 resulted in transgenic plants with an improved saccharification efficiency of both leaves and stems, which (i) was proportional to the expression level of CBM3GH5 (Figure 5B,C), and (ii) increased in transgenic plants in a temperature-dependent manner (Figure 5D), thus confirming the involvement of CBM3GH5. However, the improved saccharification yield is still too low to sustain biofuel production in a cost-effective manner; therefore, further optimizations will be required to support progress toward biofuel from plants. For example, the use of a highly thermostable CWDE blend is likely to further improve the saccharification efficiency, as suggested by the observation that biomass treatment at 70–75 °C boosted the activity of CBM3GH5 up to 40% (Figure 4D) [57]. Other strategies aimed at improving the enzyme yield of CBM3GH5 may include the use of tissue-specific and age-regulated promoters to drive the expression of PSV-targeted CWDE—i.e., by maximizing the amount of CBM3GH5 in PSVs at the desired developmental stage. Moreover, in order to explain the low yield of recombinant CBM3GH5-HA-VAC, the yield analysis of other categories of PSV-targeted CWDEs is necessary to exclude the possibility that CBM3GH5 was a particularly problematic enzyme to produce in tobacco. Lastly, opposite to the apoplastic targeting of CBM3GH5, the compartmentalized accumulation of CBM3GH5 into PSVs allowed the isolation of stable expressing plants, pointing to the PSV targeting-based technology as a valuable option when other expression strategies are not effective.

## 4. Materials and Methods

### 4.1. Synthesis In Vitro and Cloning of the Gene Encoding CBM3GH5

The cellobiohydrolase portion of CelB from *C. saccharolyticus,* formerly known as CBM3GH5 (UniprotKB: P10474, aa 380-1039) [22], was reverse-translated into the codon-optimized sequence for nuclear expression in *N. tabacum* by using the software OPTIMIZER (http://genomes.urv.es/OPTIMIZER/) [58]. The codon-optimized sequence encoding the signal peptide (SP1) of Polygalacturonase-Inhibiting Protein 2 from *Phaseolus vulgaris* (MTQFNIPVTMSSSLSIILVILVSLRTALSE) and the sequences encoding the HA-epitope (underlined amino acids) and the C-terminal Pro-Peptide of Chitinase 1 (CTPP) from *N. tabacum* (YPYDVPDYAGNGLLVDTM) were added in frame to the 5^I^ and 3^I^ ends of CBM3GH5-encoding sequence, respectively. The sequences encoding the restriction sites XbaI and SacI were added at the 5^I^ and 3^I^ ends of the sequence. The entire sequence was synthesized by GeneArt (Life Technologies, Regensburg, Germania) (Data S1). The synthetic gene was cloned downstream of the 35S promoter into the binary vector pBI121 (Clonetech) using the restriction sites XbaI and SacI. For the apoplastic CBM3GH5-HA version, the same sequence was readapted by PCR amplification using the primers Apo Fw and Apo Rv (Table S1), in order to eliminate the sequence encoding the CTPP (Data S1), and then cloned in the same vector by using the same restriction sites. *E. coli* strain XL10gold (Agilent Technologies) was transformed with these constructs and used for plasmid propagation. Sequencing of the genes was performed in order to exclude the presence of undesired mutations using the primers Apo Fw, CBH785 Fw, CBH1565 Fw and CBH240 Rv (Table S1). The CBM3GH5-HA was heterologously expressed in the *C. reinhardtii* chloroplast as reported in [31].

### 4.2. Transient and Stable Expression of CBM3GH5 in N. tabacum by Agrobacterium-Mediated Transformation

*A. tumefaciens* GV3101 and *N. tabacum* cv. Petit Havana SR1, here referred as wild type (WT), were used to generate transgenic tobacco plants. The pBI121 binary vector containing the synthetic gene was introduced into *Agrobacterium tumefaciens* strain GV3101 by electroporation. For transient expression, agroinfiltration was performed according to [59] with some modifications. Bacterial suspension in infiltration buffer (10 mM MES, 10 mM MgCl_2_, 100 µM acetosyringone, pH 5.6) at a final Abs_600_ of 0.8 was used for syringe infiltration of 6-week-old *N. tabacum* plants. Three leaves were infiltrated for each plant and for each construct. Protein extraction was performed from infiltrated leaves at 2- and 3-days post-infiltration (dpi). Stable transformation of *N. tabacum* was carried out by co-cultivating *Agrobacterium* and tobacco leaf disks according to [60,61]; about 50 leaf disks were used for each transformation cycle. Selection of transformants was performed using the appropriate concentration of plant hormones and kanamycin (150 µg mL^−1^) as selection marker. Carbenicillin (250 µg mL^−1^) and cefotaxime (250 µg mL^−1^) were added to selective medium in order to eliminate residual *Agrobacterium* cells upon the co-cultivation step.

### 4.3. Growth and Selection of Transgenic Tobacco Plants

Regenerated plants were transferred to a mist bed for a week before being moved to a bench in the greenhouse. When plants reached 15 cm in height, they were moved to 2 gal containers (7.6 L) to allow for further growth until reaching maturity. Regenerated plants were screened by PCR and subsequently by an activity assay. T1 plants that showed to be positive from the activity assay were grown until maturation and their seeds were harvested. About 100 seeds from each T1 transformant were surface-sterilized and plated on MS medium containing kanamycin as selective agent for segregation analysis. About 10–15 T2 plants from 2 independent 3:1 segregating lines (i.e., CBM3GH5-HA-VAC#4 and #6) were grown and used for further characterization.

### 4.4. Immuno-Decoration Analysis and Activity Assay Using Protein Extract from Transgenic Tobacco Plants

Protein extraction was performed by using Tween 20- and NaCl-supplemented buffers. Tween 20-supplemented buffer: 20 mM Na Citrate pH 5.5, 0.5% Tween 20. NaCl-supplemented buffer: 20 mM Na Citrate pH 5.5, NaCl 0.8 M. Leaves, stems, roots and seeds from transgenic plants or agroinfiltrated leaves were homogenized in liquid nitrogen and the resulting powders were quickly suspended in the extraction buffer at the ratio (1 mL buffer: 1 g FW grinded tissue). After optimizing the ratio [mL extraction buffer: g FW tissue], the subsequent extractions were performed by using 2 mL Tween 20-supplemented buffer per g FW tissue. Protein extraction by Tween 20-supplemented buffer was carried out by incubating the resuspended sample for 1 h at 70 °C, under gentle shaking, followed by extraction with NaCl-based buffer by incubating the resuspension for 30 min at 4 °C, under gentle shaking. After incubation, the sample was centrifuged at 14,000× *g* 10 min and the supernatant was used for downstream applications. Protein extracts from 10 mg FW of plant materials were either loaded onto SDS-PAGE for immuno-decoration analysis using a primary α-HA antibody (HA7 clone, Sigma-Aldrich) or used for enzymatic assays. Determination of the protein content was performed by Pierce™ BCA Protein Assay Kit (Thermo-Fisher Scientific). For enzymatic assays, the protein extracts (1/10 of the total reaction volume) were incubated in a buffer containing 50 mM Na Acetate pH 5.5 and 1% Azo-CM-Cellulose (Megazyme) as substrate. Alternatively, the protein extracts were dialyzed using Vivaspin 10,000 MWCO PES (Sartorius) and used for enzymatic assays using a buffer containing 50 mM Na Acetate pH 5.5 and 1% CMC (Sigma-Aldritch) as substrate. The conditions for the enzymatic reaction were set at 75 °C and pH 5.5, on the basis of the previous characterization [22]. Activity was expressed as Enzyme Units per gram dry weight (DW) tissue. Enzyme Units were expressed as µmol (reducing) ends released per minute, unless otherwise stated. Determination of µmol reducing ends released upon hydrolysis of CMC was performed according to [62] using different amounts of glucose as the calibration curve. Values of Enzyme Units were calculated as mean of two different time-points; the same reaction performed by using autoclaved extract was used as a negative control. Sampling of leaf material was performed according to the nomenclature reported in [56]. Leaves were named starting from cotyledons. Numeration started from the dicotyledonous leaves, classified as first and second leaves, respectively. For enzymatic assays, similar amount of each leaf was sampled and gathered into 3 groups: L3–L4, L5–L10, L11–L13.

### 4.5. Gene Expression Analysis in N. tabacum

Gene expression analysis of *CBM3GH5-HA-VAC* was performed on 55-day-old plants using leaves at the similar developmental stage (L5–L6). Gene expression was analysed using *EF1α* as internal reference according to [63]. Primers used for q-RT-PCR analysis are reported in Table S1 (CBHRT Fw/Rv, EFRT Fw/Rv). Experimental design and procedures were performed according to [13].

### 4.6. Purification of Recombinant Cellobiohydrolase from CBM3GH5-HA-VAC#4-11 Plants

Extraction in nondenaturing condition was performed from 5 g FW CBM3GH5-HA-VAC#4-11 leaf material, by a modified buffer (10 mM Tris-HCl pH 7.5, 0.4% Tween 20) at the optimized ratio [2 mL extraction buffer: 1 g FW leaf]. After 1 h incubation at 70 °C, the sample was centrifuged at 14,000× *g* 10 min to promote the precipitation of thermal-denaturated protein [40]. Supernatant was loaded on a Q-sepharose column (Amersham) equilibrated with 20 mM Tris-HCl pH 7.5. Elution was performed by using a stepwise NaCl gradient (from 0 to 1 M NaCl, with 0.1 M increments). Fractions eluted from the Q-sepharose column were tested by enzymatic assay and analyzed by SDS-PAGE for determination of enzyme concentration using different amount of BSA as calibration standard. Protein concentration was assessed using the Quantity-One software (Biorad). The specific activity of the enzyme (Units mg enzyme^−1^) was used for determining the amount of recombinant enzyme in CBM3GH5HAVAC#4-11 plants. Deglycosylation of purified CBM3GH5 isoforms was performed by using PNGase A according to the manufacturer’s instructions (P0707, New England Biolabs).

### 4.7. Saccharification Assay on Plant Materials

Saccharification of plant materials was performed according to [41] with some modifications. Before sampling, plants were incubated in the dark for 1 day in order to minimize the starch content. L5–L10 leaves and stems (delimited by the second and fifth internodes) were collected from 75-day-old tobacco plants. Samples were sterilized in a 1% sodium hypochlorite solution for 5 min and cut into 0.2-cm thin stripes with a razor blade; leaf and stem stripes were washed three times with sterile water. Plant samples (150–250 mg FW) were incubated in a filter-sterilized solution containing 50 mM Na Acetate pH 5.5, 0.4% (*v*/*v*) Tween-20, 0.02% (*w*/*v*) NaN_3_ and 1% (*v*/*v*) Celluclast^®^ 1.5 L (cellulase from *Trichoderma reesei* ATCC 26921). The incubation of leaf and stem stripes was carried out at either 55 or 25 °C. The enzymatic saccharification efficiency was determined as released reducing sugars vs. total sugars measured in the untreated plant material.

### 4.8. Determination of Total Carbohydrates in the Incubation Medium and in the Leaf Material

Determination of µmol reducing ends released upon hydrolysis was performed according to [62] using difference amounts of glucose as calibration curve. Total carbohydrates in plant material were determined upon acid-hydrolysis in accordance with the Laboratory Analytical Procedure of the National Renewable Energy Laboratory (https://www.nrel.gov/): sample was first hydrolyzed in 72% (*v*/*v*) sulfuric acid at 30 °C for 1 h and then in 4% (*v*/*v*) sulfuric acid at 120 °C for 1 h. Total sugars were estimated spectrophotometrically by using the phenol–sulfuric acid assay [64]. Glucose released upon the enzymatic hydrolysis was quantified by a glucose-oxidase/peroxidase assay (GOPOD assay kit, Megazyme) and expressed as released glucose vs. total sugars measured in the untreated plant material. Values are reported as mean of three different replicates.

## Supplementary Materials

The following are available online at https://www.mdpi.com/2223-7747/9/12/1799/s1. Figure S1. Presence of CBM3GH5-HA gene sequence in five representative T1 independent tobacco transformants; Figure S2. Deglycosylation of the partially purified CBM3GH5-HA-VAC by PNGase A treatment; Figure S3. Transgenic CBM3GH5-HA-VAC plants release more glucose than WT upon the enzymatic hydrolysis; Table S1. Primers used in this study; Data S1. Gene sequences encoding CBM3GH5-HA and CBM3GH5-HA-VAC. See Supplementary Material file for further details.

## References

[1] Li Q., Song J., Peng S., Wang J., Qu G.-Z., Sederoff R.R., Chiang V.L. (2014). Plant biotechnology for lignocellulosic biofuel production. Plant Biotechnol. J..

[2] Benedetti M., Locci F., Gramegna G., Sestili F., Savatin D.-V. (2019). Green production and biotechnological applications of cell wall lytic enzymes. Appl. Sci..

[3] Kubicek C.P., Starr T.L., Glass N.L. (2014). Plant cell wall–degrading enzymes and their secretion in plant-pathogenic fungi. Annu. Rev. Phytopathol..

[4] Lagaert S., Beliën T., Volckaert G. (2009). Plant cell walls: Protecting the barrier from degradation by microbial enzymes. Semin. Cell Dev. Biol..

[5] Boudart G., Charpentier M., Lafitte C., Martinez Y., Jauneau A., Gaulin E., Esquerré-Tugayé M.-T., Dumas B. (2003). Elicitor activity of a fungal endopolygalacturonase in tobacco requires a functional catalytic site and cell wall localization. Plant Physiol..

[6] Benedetti M., Pontiggia D., Raggi S., Cheng Z., Scaloni F., Ferrari S., Ausubel F.M., Cervone F., De Lorenzo G. (2015). Plant immunity triggered by engineered in vivo release of oligogalacturonides, damage-associated molecular patterns. Proc. Natl. Acad. Sci. USA.

[7] De Azevedo Souza C., Li S., Lin A.Z., Boutrot F., Grossmann G., Zipfel C., Somerville S.C. (2017). Cellulose-derived oligomers act as damage-associated molecular patterns and trigger defense-like responses. Plant Physiol..

[8] Ma Y., Han C., Chen J., Li H., He K., Liu A., Li D. (2015). Fungal cellulase is an elicitor but its enzymatic activity is not required for its elicitor activity. Mol. Plant Pathol..

[9] Poinssot B., Vandelle E., Bentéjac M., Adrian M., Levis C., Brygoo Y., Garin J., Sicilia F., Coutos-Thévenot P., Pugin A. (2003). The endopolygalacturonase 1 from *Botrytis cinerea* activates grapevine defense reactions unrelated to its enzymatic activity. Mol. Plant Microbe Interact..

[10] Choi H.W., Klessig D.F. (2016). DAMPs, MAMPs, and NAMPs in plant innate immunity. BMC Plant Biol..

[11] Frías M., González M., González C., Brito N. (2019). A 25-residue peptide from *Botrytis cinerea* xylanase BcXyn11A elicits plant defenses. Front. Plant Sci..

[12] Zipfel C. (2014). Plant pattern-recognition receptors. Trends Immunol..

[13] Benedetti M., Verrascina I., Pontiggia D., Locci F., Mattei B., De Lorenzo G., Cervone F. (2018). Four Arabidopsis berberine bridge enzyme-like proteins are specific oxidases that inactivate the elicitor-active oligogalacturonides. Plant J..

[14] Capodicasa C., Vairo D., Zabotina O., McCartney L., Caprari C., Mattei B., Manfredini C., Aracri B., Benen J., Knox J.P. (2004). Targeted modification of homogalacturonan by transgenic expression of a fungal polygalacturonase alters plant growth. Plant Physiol..

[15] Giovannoni M., Gramegna G., Benedetti M., Mattei B. (2020). Industrial use of cell wall degrading enzymes: The fine line between production strategy and economic feasibility. Front. Bioeng. Biotechnol..

[16] Petersen K., Bock R. (2011). High-level expression of a suite of thermostable cell wall-degrading enzymes from the chloroplast genome. Plant Mol. Biol..

[17] Castiglia D., Sannino L., Marcolongo L., Ionata E., Tamburino R., De Stradis A., Cobucci-Ponzano B., Moracci M., La Cara F., Scotti N. (2016). High-level expression of thermostable cellulolytic enzymes in tobacco transplastomic plants and their use in hydrolysis of an industrially pretreated *Arundo donax* L. biomass. Biotechnol. Biofuels.

[18] Harrison M.D., Geijskes J., Coleman H.D., Shand K., Kinkema M., Palupe A., Hassall R., Sainz M., Lloyd R., Miles S. (2011). Accumulation of recombinant cellobiohydrolase and endoglucanase in the leaves of mature transgenic sugar cane. Plant Biotechnol. J..

[19] Ziegelhoffer T., Will J., Austin-Phillips S. (1999). Expression of bacterial cellulase genes in transgenic alfalfa (*Medicago sativa* L.), potato (*Solanum tuberosum* L.) and tobacco (*Nicotiana tabacum* L.). Mol. Breed..

[20] Dai Z., Quesenberry R.D., Gao J., Hooker B.S. (1999). Expression of *Trichoderma reesei* exo-cellobiohydrolase I in transgenic tobacco leaves and calli. Appl. Biochem. Biotechnol..

[21] Jin S., Kanagaraj A., Verma D., Lange T., Daniell H. (2011). Release of hormones from conjugates: Chloroplast expression of β-glucosidase results in elevated phytohormone levels associated with significant increase in biomass and protection from aphids or whiteflies conferred by sucrose esters. Plant Physiol..

[22] Park J.I., Kent M.S., Datta S., Holmes B.M., Huang Z., Simmons B.A., Sale K.L., Sapra R. (2011). Enzymatic hydrolysis of cellulose by the cellobiohydrolase domain of CelB from the hyperthermophilic bacterium *Caldicellulosiruptor saccharolyticus*. Bioresour. Technol..

[23] Jiang L., Phillips T.E., Hamm C.A., Drozdowicz Y.M., Rea P.A., Maeshima M., Rogers S.W., Rogers J.C. (2001). The protein storage vacuole: A unique compound organelle. J. Cell Biol..

[24] Hinz G. (1999). Vacuolar storage proteins and the putative vacuolar sorting receptor BP-80 exit the Golgi apparatus of developing pea cotyledons in different transport vesicles. Plant Cell Online.

[25] Neuhaus J.M., Ahl-Goy P., Hinz U., Flores S., Meins F. (1991). High-level expression of a tobacco chitinase gene in *Nicotiana sylvestris*. Susceptibility of transgenic plants to *Cercospora nicotianae* infection. Plant Mol. Biol..

[26] Neuhaus J.M., Sticher L., Meins F., Boller T. (1991). A short C-terminal sequence is necessary and sufficient for the targeting of chitinases to the plant vacuole. Proc. Natl. Acad. Sci. USA.

[27] Claude S.J.D., Marie-Agnès G., Catalina R., Nadine P., Marie-Christine K.M., Jean-Marc N., Loïc F., Véronique G. (2005). Targeting of proConA to the plant vacuole depends on its nine amino-acid C-terminal propeptide. Plant Cell Physiol..

[28] Stigliano E., Di Sansebastiano G.-P., Neuhaus J.-M. (2014). Contribution of chitinase A’s C-terminal vacuolar sorting determinant to the study of soluble protein compartmentation. Int. J. Mol. Sci..

[29] D’Ovidio R., Raiola A., Capodicasa C., Devoto A., Pontiggia D., Roberti S., Galletti R., Conti E., O’Sullivan D., De Lorenzo G. (2004). Characterization of the complex locus of bean encoding polygalacturonase-inhibiting proteins reveals subfunctionalization for defense against fungi and insects. Plant Physiol..

[30] Park M., Kim S.J., Vitale A., Hwang I. (2004). Identification of the protein storage vacuole and protein targeting to the vacuole in leaf cells of three plant species. Plant Physiol..

[31] Benedetti M., Barera S., Longoni P., Guardini Z., Herrero Garcia N., Bolzonella D., Lopez-Arredondo D., Herrera-Estrella L., Goldschmidt-Clermont M., Bassi R. (2020). A microalgal-based preparation with synergistic cellulolytic and detoxifying action towards chemical-treated lignocellulose. Plant Biotechnol. J..

[32] Eamens A., Wang M.-B., Smith N.A., Waterhouse P.M. (2008). RNA silencing in plants: Yesterday, today, and tomorrow. Plant Physiol..

[33] Aziz A., Gauthier A., Bézier A., Poinssot B., Joubert J.-M., Pugin A., Heyraud A., Baillieul F. (2007). Elicitor and resistance-inducing activities of beta-1,4 cellodextrins in grapevine, comparison with beta-1,3 glucans and alpha-1,4 oligogalacturonides. J. Exp. Bot..

[34] Ferrari S., Savatin D.-V., Sicilia F., Gramegna G., Cervone F., De Lorenzo G. (2013). Oligogalacturonides: Plant damage-associated molecular patterns and regulators of growth and development. Front. Plant Sci..

[35] Lee S.C., West C.A. (1981). Polygalacturonase from *Rhizopus stolonifer*, an elicitor of casbene synthetase activity in castor bean (*Ricinus communis* L.) seedlings. Plant Physiol..

[36] Ma C.J. (2008). Cellulase elicitor induced accumulation of capsidiol in *Capsicum annumm* L. suspension cultures. Biotechnol. Lett..

[37] Hraška M., Rakouský S., Čurn V. (2008). Tracking of the CaMV-35S promoter performance in GFP transgenic tobacco, with a special emphasis on flowers and reproductive organs, confirmed its predominant activity in vascular tissues. Plant Cell Tissue Organ Cult..

[38] Harrison M.D., Geijskes R.J., Lloyd R., Miles S., Palupe A., Sainz M.B., Dale J.L. (2014). Recombinant cellulase accumulation in the leaves of mature, vegetatively propagated transgenic sugarcane. Mol. Biotechnol..

[39] Herman E., Larkins B. (1999). Protein storage bodies and vacuoles. Plant Cell.

[40] Patchett M.L., Neal T.L., Schofield L.R., Strange R.C., Daniel R.M., Morgan H.W. (1989). Heat treatment purification of thermostable cellulase and hemicellulase enzymes expressed in *E. coli*. Enzym. Microb. Technol..

[41] Lionetti V., Francocci F., Ferrari S., Volpi C., Bellincampi D., Galletti R., D’Ovidio R., De Lorenzo G., Cervone F. (2010). Engineering the cell wall by reducing de-methyl-esterified homogalacturonan improves saccharification of plant tissues for bioconversion. Proc. Natl. Acad. Sci. USA.

[42] Gama R., Van Dyk J.S., Pletschke B.I. (2015). Optimisation of enzymatic hydrolysis of apple pomace for production of biofuel and biorefinery chemicals using commercial enzymes. 3 Biotech.

[43] Weng J.K., Li X., Bonawitz N.D., Chapple C. (2008). Emerging strategies of lignin engineering and degradation for cellulosic biofuel production. Curr. Opin. Biotechnol..

[44] Bindschedler L.V., Tuerck J., Maunders M., Ruel K., Petit-Conil M., Danoun S., Boudet A.M., Joseleau J.P., Bolwell G.P. (2007). Modification of hemicellulose content by antisense down-regulation of UDP-glucuronate decarboxylase in tobacco and its consequences for cellulose extractability. Phytochemistry.

[45] Biswal A.K., Atmodjo M.A., Li M., Baxter H.L., Yoo C.G., Pu Y., Lee Y., Mazarei M., Black I.M., Zhang J. (2018). Sugar release and growth of biofuel crops are improved by downregulation of pectin biosynthesis. Nat. Biotechnol..

[46] Greene E.R., Himmel M.E., Beckham G.T., Tan Z. (2015). Glycosylation of cellulases. Adv. Carbohydr. Chem. Biochem..

[47] Marin Viegas V.S., Ocampo C.G., Petruccelli S. (2017). Vacuolar deposition of recombinant proteins in plant vegetative organs as a strategy to increase yields. Bioengineered.

[48] Klose H., Günl M., Usadel B., Fischer R., Commandeur U. (2013). Ethanol inducible expression of a mesophilic cellulase avoids adverse effects on plant development. Biotechnol. Biofuels.

[49] Zuo J., Niu Q.-W., Chua N.-H. (2000). An estrogen receptor-based transactivator XVE mediates highly inducible gene expression in transgenic plants. Plant J..

[50] Tomassetti S., Pontiggia D., Verrascina I., Reca I.B., Francocci F., Salvi G., Cervone F., Ferrari S. (2015). Controlled expression of pectic enzymes in *Arabidopsis thaliana* enhances biomass conversion without adverse effects on growth. Phytochemistry.

[51] Mir B.A., Mewalal R., Mizrachi E., Myburg A.A., Cowan D.A. (2014). Recombinant hyperthermophilic enzyme expression in plants: A novel approach for lignocellulose digestion. Trends Biotechnol..

[52] Mir B.A., Myburg A.A., Mizrachi E., Cowan D.A. (2017). In planta expression of hyperthermophilic enzymes as a strategy for accelerated lignocellulosic digestion. Sci. Rep..

[53] Sarmiento F., Peralta R., Blamey J.M. (2015). Cold and hot extremozymes: Industrial relevance and current trends. Front. Bioeng. Biotechnol..

[54] Poole D.M., Morag E., Lamed R., Bayer E.A., Hazlewood G.P., Gilbert H.J. (1992). Identification of the cellulose-binding domain of the cellulosome subunit S1 from *Clostridium thermocellum* YS. FEMS Microbiol. Lett..

[55] Carrard G., Koivula A., Soderlund H., Beguin P. (2000). Cellulose-binding domains promote hydrolysis of different sites on crystalline cellulose. Proc. Natl. Acad. Sci. USA.

[56] Faè M., Accossato S., Cella R., Fontana F., Goldschmidt-Clermont M., Leelavathi S., Reddy V.S., Longoni P. (2017). Comparison of transplastomic *Chlamydomonas reinhardtii* and *Nicotiana tabacum* expression system for the production of a bacterial endoglucanase. Appl. Microbiol. Biotechnol..

[57] Benedetti M., Vecchi V., Betterle N., Natali A., Bassi R., Dall’Osto L. (2019). Design of a highly thermostable hemicellulose-degrading blend from *Thermotoga neapolitana* for the treatment of lignocellulosic biomass. J. Biotechnol..

[58] Puigbò P., Guzmán E., Romeu A., Garcia-Vallvé S. (2007). OPTIMIZER: A web server for optimizing the codon usage of DNA sequences. Nucleic Acids Res..

[59] Yang Y., Li R., Qi M. (2000). In vivo analysis of plant promoters and transcription factors by agroinfiltration of tobacco leaves. Plant J..

[60] Horsch R.B., Fry J.E., Hoffmann N.L., Eichholtz D., Rogers S.G., Fraley R.T. (1985). A simple and general method for transferring genes into plants. Science.

[61] Rogers S.G., Horsch R.B., Fraley R.T. (1986). Gene transfer in plants: Production of transformed plants using Ti plasmid vectors. Methods Enzymol..

[62] Lever M. (1972). A new reaction for colorimetric determination of carbohydrates. Anal. Biochem..

[63] Schmidt G.W., Delaney S.K. (2010). Stable internal reference genes for normalization of real-time RT-PCR in tobacco (*Nicotiana tabacum*) during development and abiotic stress. Mol. Genet. Genomics.

[64] Dubois M., Gilles K.A., Hamilton J.K., Rebers P.A., Smith F. (1956). Colorimetric method for determination of sugars and related substances. Anal. Chem..

