# Supplementary material for: Expression of a Hyperthermophilic Cellobiohydrolase in Transgenic Nicotiana tabacum by Protein Storage Vacuole Targeting
Source: Plants (Basel). 2020 Dec 18;9(12):1799. doi: 10.3390/plants9121799 (PMC7767180; doi:10.3390/plants9121799)
Supplement: Supplementary file 1 [file plants-09-01799-s001.pdf]

Supplementary Materials

Figure S1.

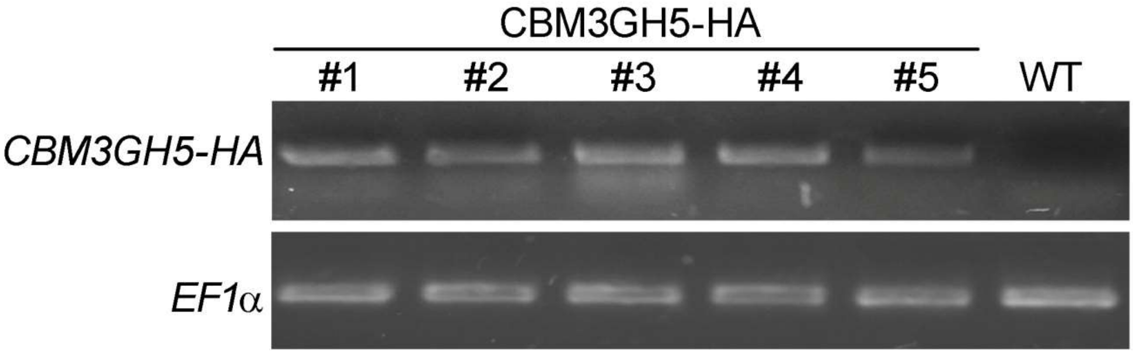

Figure S1. Presence of *CBM3GH5-HA* gene sequence in five representative T1 independent tobacco transformants. PCR analysis of *CBM3GH5-HA* was performed using 0.5 μg of gDNA from five T1 transformants. PCR analysis using 0.5 μg gDNA from WT plants was used as negative control. Amplification of *EF1α* (Eukaryotic Translation Elongation Factor 1 alpha) was used as internal reference.

Figure S2

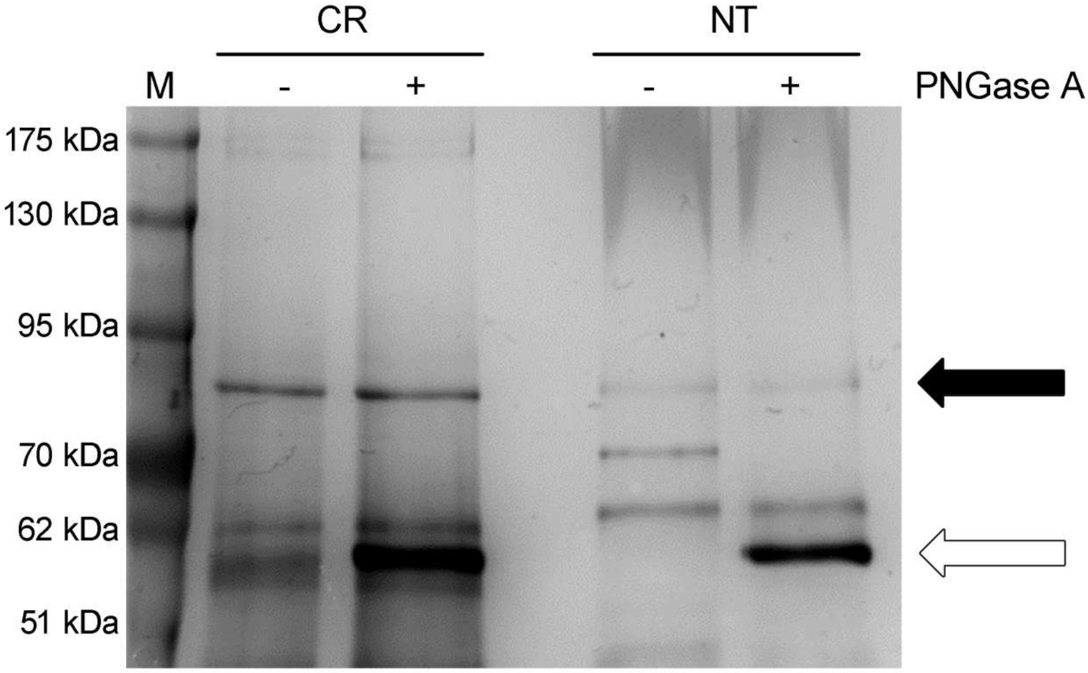

Figure S2. Deglycosylation of the partially purified CBM3GH5-HA-VAC by PNGase A treatment. SDS-PAGE analysis of fractions from AEC, before (-) and after (+) treatment with PNGase A. Analysis of Fx3 (see Figure 4b) is reported as representative result. Analysis of recombinant CBM3GH5-HA from *C. reinhardtii* (CR) is reported as control. Black and white arrow point to CBM3GH5-HA and PNGase A, respectively.

Figure S3

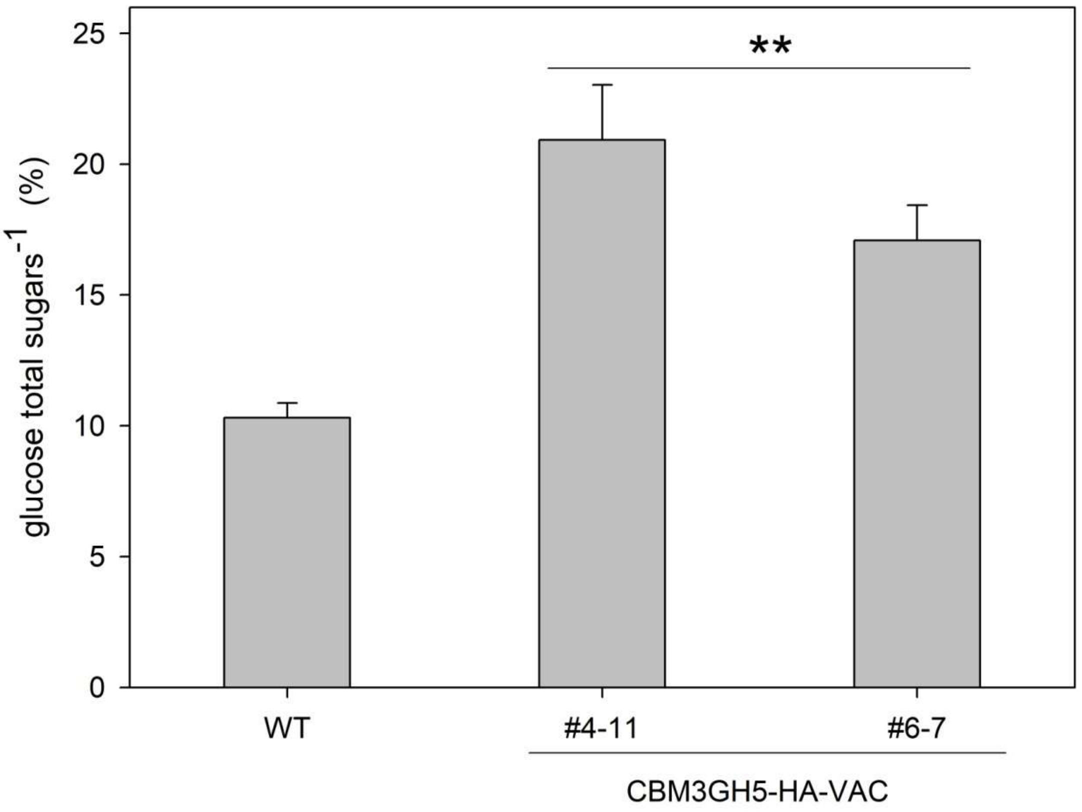

Figure S3. Transgenic CBM3GH5-HA-VAC plants release more glucose than WT upon the enzymatic hydrolysis. Glucose released from leaf tissues in the incubation medium by Celluclast treatment, upon 48 h of reaction at 55°C, as determined by GO-POD assay. The percentage is expressed as ratio between released glucose *vs.* total sugars measured in the untreated plant material. Data are expressed as mean ± SD ($n \geq 3$). Asterisks indicate statistically significant difference against control (WT) according to Student's t test (**, $P < 0.01$).

Table S1. Primers used in this study. Primers used for the construction of the expression cassette CBM3GH5-HA in *N. tabacum* (Apo Fw, Apo Rv), gene sequencing (CBH785 Fw, CBH1565 Fw, CBH240 Rv) and gene expression analysis (CBHRT Fw, CBHRT Rv, EFRT Fw, EFRT Rv) are reported.

PRIMERS	SEQUENCE
Apo Fw	CTAAGTCTAGATGACTCAATTTAATATTCC
Apo Rv	CTAAGGAGCTCTTAAGCATAATCTGGAACATCATATG
CBH785 Fw	CCAGATGATACTAATGATGATTGGC
CBH1565 Fw	TGGGGAGGAAATCTTAGAGG
CBH240 Rv	CCAGTAGCTGGAGTAGAAACTGG
CBHRT Fw	GCTGGACAACTTCAACCAGG
CBHRT Rv	AGCCAAGACCAATCATTTCCT
EFRT Fw	GGTATCCGCTCCCAGAGTT
EFRT Rv	TTCTGAGCCACCTTGGAAAAC

Data S1. Gene sequences encoding CBM3GH5-HA and CBM3GH5-HA-VAC. Codon-optimized sequence of (**A**) *CBM3GH5-HA* and (**B**) *CBM3GH5-HA-VAC* used for the nuclear expression in *Nicotiana tabacum*. Underlined sequences: restriction sites used for cloning; green sequence: START codon; yellow sequence: signal peptide of Polygalacturonase Inhibiting Protein 2 from *Phaseolus vulgaris* encoding sequence; grey sequence: CBM3GH5 from *Caldicellulosiruptor saccharolyticus* encoding sequence; blue sequence: HA epitope encoding sequence; turquoise sequence: C-Terminal Pro-Peptide of Chitinase 1 from *N. tabacum* encoding sequence; red sequence: STOP codon.

A

TCTAGATGACTCAATTTAATATTCCAGTTACTATGTCTTCTTCTCTTTCTATTATTCTT
GTTATTCTTGTTTCTCTTAGAACTGCTCTTTCTGAAGGAGTTACTACTTCTTCTCC
AACTCCAACTCCAACTCCAACTGTTACTGTTACTCCAACTCCAACTCCAACTCCA
ACTCCAACTGTTACTGCTACTCCAACTCCAACTCCAACTCCAGTTTCTACTCCAG
CTACTGGAGGACAAATTAAGGTTCTTTATGCTAATAAGGAAACTAATTCTACTA
CTAATACTATTAGACCATGGCTTAAGGTTGTTAATTCTGGATCTTCTTCTATTGA
TCTTTCAAGGGTTACTATTAGATATTGGTATACTGTTGATGGAGAAAGGGCACA
ATCTGCTGTTTCTGATTGGGCTCAAATTGGAGCTTCTAATGTTACTTTTAAGTTT
GTTAAACTTTCTTCTTCTGTTTCTGGAGCTGATTATTATCTTGAAATTGGATTTAA
GTCTGGAGCTGGACAACTTCAACCAGGAAAGGATACTGGAGAAATTCAAATTA
GATTTAATAAGTCTGATTGGTCTAATTATAATCAAGGAAATGATTGGTCTTGGCT
TCAATCTATGACTTCTTATGGAGAAAATGAAAAGGTTACTGCTTATATTGATGG
AGTTCTTGTTTGGGGACAAGAACCATCTGGAGCTACTCCAGCTCCAACTATGAC
TGTTGCTCCAACTGCTACTCCAACTCCAACTCTTTCTCCAACTGTTACTCCAACT
CCAGCTCCAACTCAAACTGCTATTCCAACTCCAACTCTTACTCCAAATCCAACTC
CAACTTCTTCTATTCCAGATGATACTAATGATGATTGGCTTTATGTTTCTGGAAA
TAAGATTGTTGATAAGGATGGAAGACCAGTTTGGCTTACTGGAATTAATTGGTT
TGGATATAATACTGGAACTAATGTTTTTGATGGAGTTTGGTCTTGTAATCTTAAG
GATACTCTTGCTGAAATTGCTAATAGAGGATTTAATCTTCTTAGAGTTCCAATTT
CTGCTGAACTTATTCTTAATTGGTCTCAAGGAATTTATCCAAAGCCAAATATTAA
TTATTATGTTAATCCAGAACTTGAAGGAAAGAATTCTCTTGAAGTTTTTGATATT
GTTGTTCAAACTTGTAAGGAAGTTGGACTTAAGATTATGCTTGATATTCATTCTA
TTAAGACTGATGCTATGGGACATATTTATCCAGTTTGGTATGATGAAAAGTTTAC
TCCAGAAGATTTTTATAAGGCTTGTGAATGGATTACTAATAGATATAAGAATGA
TGATACTATTATTGCTTTTGATCTTAAGAATGAACCACATGGAAAGCCATGGCA
AGATACTACTTTTGCTAAGTGGGATAATTCTACTGATATTAATAATTGGAAGTAT
GCTGCTGAAACTTGTGCTAAGAGAATTCTTAATATTAATCCAAATCTTCTTATTG
TTATTGAAGGAATTGAAGCATATCCAAAGGATGATGTTACTTGGACTTCTAAGT
CTTCTTCTGATTATTATTCTACTTGGTGGGGAGGAAATCTTAGAGGAGTTAGAAA
GTATCCAATTAATCTTGGAAAGTATCAAAATAAGGTTGTTTATTCTCCACATGAT
TATGGACCATCTGTTTATCAACAACCATGGTTTTATCCAGGATTTACTAAGGAAT
CTCTTCTTCAAGATTGTTGGAGACCAAATTGGGCTTATATTATGGAAGAAAATA
TTGCTCCACTTCTTATTGGAGAATGGGGAGGACATCTTGATGGAGCTGATAATG
AAAAGTGGATGAAGTATCTTAGAGATTATATTATTGAAAATCATATTCATCATA
CTTTTTGGTGTTTTAATGCTAATTCTGGAGATACTGGAGGACTTGTTGGATATGA

TTTTACTACTTGGGATGAAAAGAAGTATTCTTTTCTTAAGCCAGCTCTTTGGCAA
GATTCTCAAGGAAGATTTGTTGGACTTGATCATAAGAGACCACTTGGAACTAAT
GGAAAGAATATTAATATTACTACTTATTATAATAATAATGAACCAGAACCAGTT
CCAGCTTCTAAGTATCCATATGATGTTCCAGATTATGCTTAAGAGCTC

B

TCTAGATGACTCAATTTAATATTCCAGTTACTATGTCTTCTTCTCTTTCTATTATTCTT
GTTATTCTTGTTTCTCTTAGAACTGCTCTTTCTGAAGGAGTTACTACTTCTTCTCC
AACTCCAACTCCAACTCCAACTGTTACTGTTACTCCAACTCCAACTCCAACTCCA
ACTCCAACTGTTACTGCTACTCCAACTCCAACTCCAACTCCAGTTTCTACTCCAG
CTACTGGAGGACAAATTAAGGTTCTTTATGCTAATAAGGAAACTAATTCTACTA
CTAATACTATTAGACCATGGCTTAAGGTTGTTAATTCTGGATCTTCTTCTATTGA
TCTTTCAAGGGTTACTATTAGATATTGGTATACTGTTGATGGAGAAAGGGCACA
ATCTGCTGTTTCTGATTGGGCTCAAATTGGAGCTTCTAATGTTACTTTTAAGTTT
GTTAAACTTTCTTCTTCTGTTTCTGGAGCTGATTATTATCTTGAAATTGGATTTAA
GTCTGGAGCTGGACAACTTCAACCAGGAAAGGATACTGGAGAAATTCAAATTA
GATTTAATAAGTCTGATTGGTCTAATTATAATCAAGGAAATGATTGGTCTTGGCT
TCAATCTATGACTTCTTATGGAGAAAATGAAAAGGTTACTGCTTATATTGATGG
AGTTCTTGTTTGGGGACAAGAACCATCTGGAGCTACTCCAGCTCCAACTATGAC
TGTTGCTCCAACTGCTACTCCAACTCCAACTCTTTCTCCAACTGTTACTCCAACT
CCAGCTCCAACTCAAACTGCTATTCCAACTCCAACTCTTACTCCAAATCCAACTC
CAACTTCTTCTATTCCAGATGATACTAATGATGATTGGCTTTATGTTTCTGGAAA
TAAGATTGTTGATAAGGATGGAAGACCAGTTTGGCTTACTGGAATTAATTGGTT
TGGATATAATACTGGAACTAATGTTTTTGATGGAGTTTGGTCTTGTAATCTTAAG
GATACTCTTGCTGAAATTGCTAATAGAGGATTTAATCTTCTTAGAGTTCCAATTT
CTGCTGAACTTATTCTTAATTGGTCTCAAGGAATTTATCCAAAGCCAAATATTAA
TTATTATGTTAATCCAGAACTTGAAGGAAAGAATTCTCTTGAAGTTTTTGATATT
GTTGTTCAAACTTGTAAGGAAGTTGGACTTAAGATTATGCTTGATATTCATTCTA
TTAAGACTGATGCTATGGGACATATTTATCCAGTTTGGTATGATGAAAAGTTTAC
TCCAGAAGATTTTTATAAGGCTTGTGAATGGATTACTAATAGATATAAGAATGA
TGATACTATTATTGCTTTTGATCTTAAGAATGAACCACATGGAAAGCCATGGCA
AGATACTACTTTTGCTAAGTGGGATAATTCTACTGATATTAATAATTGGAAGTAT
GCTGCTGAAACTTGTGCTAAGAGAATTCTTAATATTAATCCAAATCTTCTTATTG
TTATTGAAGGAATTGAAGCATATCCAAAGGATGATGTTACTTGGACTTCTAAGT
CTTCTTCTGATTATTATTCTACTTGGTGGGGAGGAAATCTTAGAGGAGTTAGAAA
GTATCCAATTAATCTTGGAAAGTATCAAAATAAGGTTGTTTATTCTCCACATGAT
TATGGACCATCTGTTTATCAACAACCATGGTTTTATCCAGGATTTACTAAGGAAT
CTCTTCTTCAAGATTGTTGGAGACCAAATTGGGCTTATATTATGGAAGAAAATA
TTGCTCCACTTCTTATTGGAGAATGGGGAGGACATCTTGATGGAGCTGATAATG
AAAAGTGGATGAAGTATCTTAGAGATTATATTATTGAAAATCATATTCATCATA
CTTTTTGGTGTTTTAATGCTAATTCTGGAGATACTGGAGGACTTGTTGGATATGA
TTTTACTACTTGGGATGAAAAGAAGTATTCTTTTCTTAAGCCAGCTCTTTGGCAA
GATTCTCAAGGAAGATTTGTTGGACTTGATCATAAGAGACCACTTGGAACTAAT

GGAAAGAATATTAATATTACTACTTATTATAATAATAATGAACCAGAACCAGTT
CCAGCTTCTAAGTATCCATATGATGTTCCAGATTATGCTGGAAATGGACTTCTTG
TTGATACTATGTAA<u>GAGCTC</u>
